# “Ammonia Induced Framework Transformations and Spin-Crossover in Fe(II) Hofmann MOFs”

**DOI:** 10.1021/acs.inorgchem.6c00626

**Published:** 2026-04-01

**Authors:** Mario Pacheco, Annena Jesuman, Higinio Maqueda-Márquez, Javier González-Platas, Ana Belén Gaspar

**Affiliations:** † Institut de Ciència Molecular (ICMol)-Departament de Química Inorgànica, 16781 Universitat de València, C/Catedrático José Beltrán 2, 46980 Paterna, Spain; ‡ Departamento de Física-Instituto Universitario de Estudios Avanzados en Física Atómica, 16749 Molecular y Fotónica (IUDEA). MALTA Consolider Team. Universidad de La Laguna, Avda. Astrofísico Fco. Sánchez s/n, La Laguna, Tenerife E-38204, Spain; § Facultad de Química, Universidad de la República, Av. Gral. Flores 2124, 11800 Montevideo, Uruguay

## Abstract

Hofmann-type Fe­(II) frameworks, [Fe­(pz)­M­(CN)_4_] (pz = pyrazine; M = Pd, Pt), exhibit a dual-mode response to ammonia, arising from its strong Lewis basicity. Short-term NH_3_ exposure produces reversible clathrates (**1-Pd@NH**_**3**_, **1-Pt@NH**_**3**_) that change from orange to pale yellow, with the parent framework preserved. In these clathrates, spin-crossover behavior is strongly modulated, with transition temperatures shifted by up to 150 K and partial stabilization of the high-spin state. Prolonged exposure induces irreversible pyrazine-to-ammonia substitution at Fe­(II) axial sites, forming red, diamagnetic phases ([Fe­(NH_3_)_2_M­(CN)_4_]·2H_2_O; M = Pd (**2**), Pt (**3**)). Substitution kinetics are metal-dependent: the Pd framework transforms rapidly (∼1 h), consistent with slightly longer Pd–C/N bonds and more labile Fe–N­(pz) coordination, whereas the Pt framework transforms more slowly (∼24 h), correlating with shorter Pt–C/N bonds and higher lattice rigidity. Other polar guests (H_2_O, alcohols, pyridine) induce only reversible clathrate formation, highlighting the unique chemical reactivity of NH_3_. These results establish clear links between guest Lewis basicity, metal–ligand covalency, framework stability, and functional response, demonstrating how host–guest chemistry can be leveraged to precisely tune structural, electronic, and magnetic properties in heterometallic coordination frameworks.

## Graphical abstract

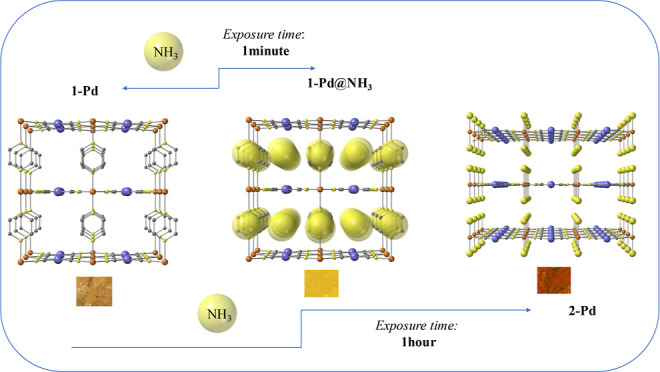

## Introduction

Metal–organic frameworks (MOFs) are a versatile class of crystalline materials, distinguished by their high porosity and tunable architectures. Constructed from metal nodes and organic linkers, MOFs combine large internal surface areas with diverse chemical functionalities, enabling applications ranging from gas storage and separation to catalysis and molecular recognition.^1–7^ Among these, Fe­(II)-based MOFs are particularly notable for their spin-crossover (SCO) behavior, in which the electronic states of the metal centers respond to external stimuli such as temperature, pressure, or light.^8–14^ This switching is accompanied by pronounced changes in magnetic, optical, and structural properties, making SCO systems a rich playground for investigating structure–property relationships.^13,14^

Bimetallic Hofmann-type^15,16^ Fe­(II) MOFs, {Fe­(L)_2_[M­(CN)_4_]} (L = pyridine-type ligand; M­(II) = Ni, Pd, Pt), represent a benchmark family of SCO frameworks.^17,18^ When a bifunctional ligand such as pyrazine (pz) pillars the 2D {Fe–[M­(CN)_4_]}∞ layers, robust 3D networks are formed, enhancing cooperativity and shifting the spin transition toward room temperature for Pd and Pt derivatives.^18,19^ The platinum analogue, [Fe­(pz)­Pt­(CN)_4_] (**1-Pt**), exhibits a sharp, hysteretic SCO near 300 K and a porous structure exquisitely sensitive to guest molecules, enabling reversible spin switching by chemical inclusion.^19^ In contrast, the palladium analogue, [Fe­(pz)­Pd­(CN)_4_] (**1-Pd**), has remained poorly characterized, with no single-crystal structure reported. This lack of atomic-level information has hindered understanding of the Fe­(II) coordination environment and subtle structural differences relative to **1-Pt**. Furthermore, the interaction of these frameworks with ammonia—a small, strongly coordinating ligand—remains largely unexplored. Ammonia can act both as a pore-filling guest and a coordinating ligand, offering a chemically informative probe of framework dynamics.

In this work, we investigate the Hofmann-type Fe­(II) frameworks [Fe­(pz)­M­(CN)_4_] (M = Pd, Pt) and their response to ammonia, a small and strongly coordinating Lewis base that has not been systematically studied in these materials. We report the first single-crystal X-ray structures of the palladium analogue, **1-Pd**, in both high-spin and low-spin states, establishing a key structural benchmark. NH_3_ interactions with **1-Pd** and **1-Pt** were studied using time-resolved spectroscopic, diffraction, and magnetic measurements. Short NH_3_ exposure produces reversible host–guest inclusion. The frameworks change color from orange to pale yellow (Figure S1). Spin-crossover behavior is strongly modulated, with transition temperatures shifted and partial stabilization of the high-spin state. Prolonged NH_3_ exposure triggers irreversible, crystallinity-retaining pyrazine-to-ammonia ligand substitution. The resulting frameworks [Fe­(NH_3_)_2_M­(CN)_4_]·2H_2_O (M = Pd (**2**) and **3**) are red and diamagnetic. These findings show that ammonia drives distinct structural, optical, and electronic responses in Fe­(II) Hofmann frameworks.

## Results and Discussion

### Synthesis and Structural Characterization

The slow-diffusion synthesis yielded high-quality orange needle-like single crystals of **1-Pd**, displaying the characteristic color of the high-spin (HS, *S* = 2) Fe­(II) state at ambient temperature (Figure S2). Single-crystal X-ray diffraction at 294 and 230 K provides the first definitive structure of this compound, which crystallizes in the tetragonal space group *P*4/*mmm*. The structure consists of 2D square-grid layers of {-Fe-[Pd­(CN)_4_]-}_∞_ in the ab-plane, pillared along the *c*-axis by trans-coordinating pyrazine ligands to form a 3D Hofmann-like network (Figure 1, left). The Fe­(II) center is in a slightly distorted octahedral [FeN_6_] environment, coordinated by four equatorial nitrogen atoms from cyanide ligands and two axial nitrogen atoms from pyrazine linkers. Key bond distances are Fe–N­(pz) = 2.229 Å, Fe–NC = 2.123 Å, Pd–CN = 1.996 Å, C–N = 1.144 Å, C–N­(pz) = 1.319 Å, and C–C­(pz) = 1.378 Å.

**Figure 1:**
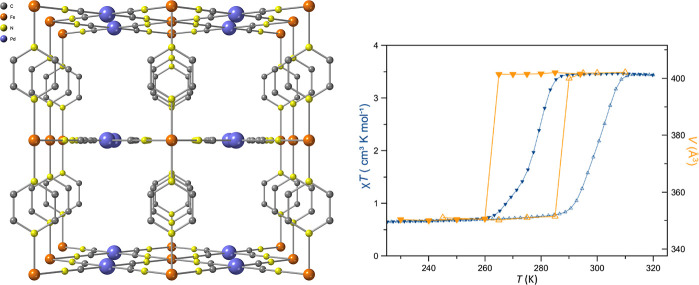
Left: view of the 3D crystal structure of **1-Pd** at 294 K along the crystallographic *b*-axis, showing the porous channels formed by the pillaring of {-Fe-[Pd­(CN)_4_]-}_∞_ layers with pyrazine ligands. Right: thermal variation of the unit cell volume from multitemperature single-crystal X-ray determination and the temperature variation of the molar magnetic susceptibility product, χ_M_ *T* vs *T*.

These Fe–N distances are unequivocally characteristic of HS Fe­(II) and are slightly longer than those typically observed in low-spin (LS) complexes (∼2.0 Å). The pyrazine pillar connects consecutive layers along the (001) direction, forming channels. The resulting porous structure exhibits an accessible void volume of 391.03 Å^3^ in the HS state, which decreases to 345.06 Å^3^ in the LS state, corresponding to a retention of ca. 88% of the original pore volume upon spin crossover. Relevant crystallographic data are reported in Tables S1–S3.

The thermal variation of the unit-cell volume, derived from multitemperature single-crystal X-ray diffraction, is shown in Figure 1 alongside the molar magnetic susceptibility product, χ_M_ *T*, measured as a function of temperature. At 320 K, the unit-cell volume of 402 Å^3^ and χ_M_ *T* ≈ 3.6 cm^3^ K mol^–1^ confirm that Fe­(II) ions are in the HS state. Upon cooling, the unit-cell volume exhibits an abrupt contraction between 260 and 270 K, whereas the magnetic susceptibility decreases more gradually over the 260–285 K interval, reflecting the progressive nature of the spin-state conversion and the known decoupling between structural and magnetic order parameters in cooperative SCO frameworks. At 220 K, both unit-cell volume and χ_M_ *T* reach stable values, evidencing complete conversion to the LS state. Upon warming, the cell volume and lattice parameters (*a*–*c*, Figure S3) display a thermal hysteresis of ∼25 K, in agreement with the magnetic measurements, confirming the cooperative and bistable nature of the transition.

The nearly constant *a*–*c* lattice parameters over 325–265 K (HS) and 265–220 K (LS) demonstrate minimal thermal expansion, with significant changes occurring only near the spin-transition temperatures. The total change in unit-cell volume associated with the SCO is 52 Å^3^ per formula unit, within the typical range for Fe­(II) systems (15–50 Å^3^). Illustrations of the reversible expansion–contraction of the porous framework are shown in Figures S4 and S5.

Notably, the structural transition appears to occur at slightly lower temperatures than the magnetic transition. This is attributed to X-ray-induced excited spin-state trapping (XIESST),^25,26^ in which the X-ray beam partially excites LS Fe­(II) centers back to the HS state during single-crystal diffraction, requiring lower temperatures to achieve complete LS conversion. In contrast, SQUID measurements are solely thermally driven. This atomic-resolution structure establishes a precise benchmark for the **1-Pd** framework, enabling quantitative evaluation of structural changes induced by external stimuli such as temperature, pressure, or guest sorption. While highly similar to the reported **1-Pt** analogue, minor differences in Fe–N bond lengths reflect subtle framework-dependent effects.

### Dual-Mode Response to Ammonia

Reversible host–guest inclusion and irreversible crystallinity-retaining ligand substitution.

### Reversible host–guest Inclusion

The pristine **1-Pd** and **1-Pt** frameworks were isolated as dihydrates and dehydrated prior to guest uptake experiments, see the experimental section for details. TGA confirms two lattice water molecules per formula unit and reveals distinct thermal stabilities (**1-Pd** ∼260 °C; **1-Pt** ∼220 °C, Figure S6). FT-IR spectra (ν­(C≡N) > 2200 cm^–1^ and pyrazine bands, 1400–700 cm^–1^, Figure S7) and PXRD patterns (Figure S8) confirm that the anhydrous frameworks are isostructural, phase-pure, and structurally robust, suitable for ammonia-responsive studies. The guest-free porous frameworks of **1-Pd** and **1-Pt** readily adsorb ammonia from the gas phase, forming the pale yellow clathrates **1-Pd@NH**_**3**_ and **1-Pt@NH**_**3**_, as described in the experimental section.

Thermogravimetric and elemental analyses suggest the formula [Fe­(pz)­M­(CN)_4_]·2NH_3_ for the palladium and platinum clathrates (Figure S9). The successful and reversible inclusion of ammonia is confirmed by FT-IR spectroscopy (Figure 2(b)). In comparison to **1-Pd** and **1-Pt**, the IR spectra of **1-Pd@NH**_**3**_ and **1-Pt@NH**_**3**_ show the emergence of the characteristic NH_3_ symmetric bending mode at approximately 1250 cm^–1^. More significantly, a substantial redshift of the framework’s cyanide stretching frequency (ν­(C≡N)) is observed, from 2220 cm^–1^ in pristine **1-Pd** and **1-Pt** to approximately 2180 cm^–1^ in **1-Pd@NH**_**3**_ and **1-Pt@NH**_**3**_. This significant shift of 40 cm^–1^ is direct spectroscopic evidence of a strong host–guest interaction, likely arising from hydrogen bonding between guest ammonia molecules and the nitrogen atoms of the cyanide ligands. Moreover, this interaction perturbs the electronic structure of the cyanide bridge, weakening the C≡N triple bond and directly altering the ligand field strength experienced by the Fe­(II) SCO center (vide infra). PXRD analysis confirms that this guest inclusion proceeds without loss of bulk crystallinity, as the primary Bragg reflections of the parent framework are retained in **1-Pd@NH**_**3**_ and **1-Pt@NH**_**3**_. (Figure 2a). The ammonia can be completely desorbed by heating the clathrates at 160 °C during 2 h. The reversibility of NH_3_ uptake was demonstrated by IR, PXRD, and magnetic measurements of the desorbed frameworks, which closely match the spectra, patterns, and magnetic properties of the pristine materials (Figure S10), confirming full structural and functional recovery.

**Figure 2:**
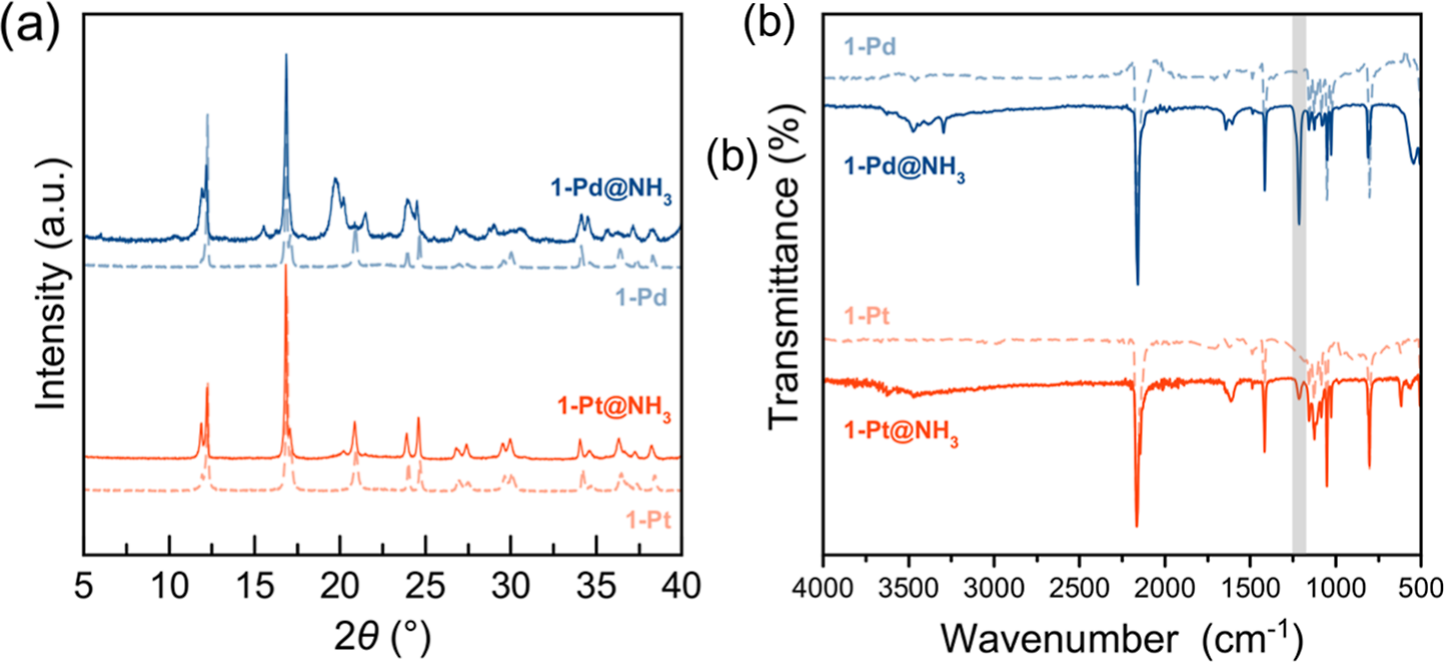
(a) Experimental PXRD patterns at 293 K for **1-Pd**@NH_3_ and **1-Pt**@NH_3_, compared with the pattern calculated from the single-crystal structure of **1-Pd** and **1-Pt** in the HS state, confirming the retention of crystallinity upon guest inclusion. (b) FT-IR spectra for pristine **1-Pd** and **1-Pt** and the ammonia clathrates **1-Pd**@NH_3_ and **1-Pt**@NH_3_. The plot highlights the significant redshift in the v­(C≡N) region, and the appearance of the characteristic NH_3_ bending mode around 1250 cm^–1^.

### Irreversible Crystallinity-Retaining Ligand Substitution

Time-dependent studies of NH_3_ uptake reveal the chemical lability of the **1-Pd** and **1-Pt** frameworks. Short exposures (∼1 min) allow reversible adsorption and modulation of the spin-crossover properties, forming the pale-yellow clathrates **1-Pd@NH**_**3**_ and **1-Pt@NH**_**3**_. Prolonged exposure (>30 min) induces an irreversible structural and chemical transformation of **1-Pd** and **1-Pt** into new two-dimensional frameworks, formulated as [Fe­(NH_3_)_2_Pd­(CN)_4_]·2H_2_O (**2**) and [Fe­(NH_3_)_2_Pt­(CN)_4_]·2H_2_O (**3**), respectively. The color of the frameworks changes from orange to red during this transformation.

Time-resolved FT-IR and PXRD measurements (Figures 3, S11) capture the transformation. FT-IR spectra show the progressive attenuation of pyrazine-associated bands (1150–1000 cm^–1^) over time, concurrent with the emergence of N–H vibrations (∼1250 cm^–1^) from coordinated ammonia. PXRD patterns evolve from the original 3D framework to a new set of well-defined reflections, indicating the formation of a distinct crystalline phase. These data collectively confirm that NH_3_ displaces pyrazine ligands from the Fe­(II) axial sites, reorganizing the 3D network into a 2D material. Attempts to collect single-crystal X-ray diffraction data were unsuccessful due to partial loss of crystal quality during NH_3_ exposure.

**Figure 3:**
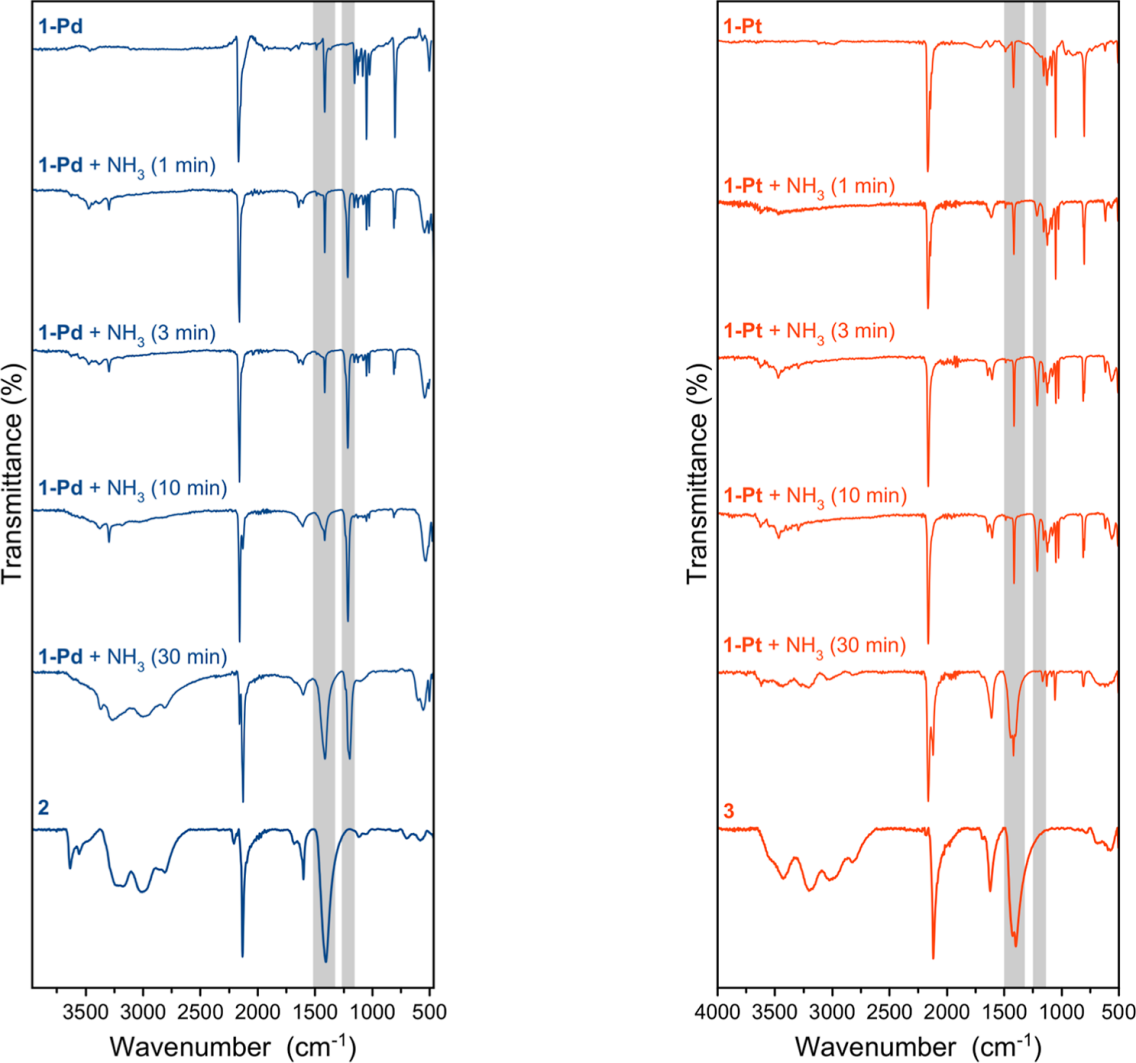
Time-resolved FT-IR showing NH_3_-induced transformation of **1-Pd** (fast) and **1-Pt** (slow). Pyrazine bands disappear as new N–H bands appear. Partial loss of crystallinity prevented single-crystal X-ray analysis; transformations retain crystallinity but are not single-crystal-to-single-crystal.

The Pd framework undergoes nearly complete conversion within 30 min, whereas the Pt analogue transforms more slowly, reaching full conversion only after ∼24 h (Figure 3). The progressive color change mirrors this behavior: both frameworks develop a pale-yellow tint initially, and gradually turn red as the irreversible phases **2** and **3** form (Figure S12). The kinetics of ligand substitution correlate with structural features: in the Pd framework, slightly longer Pd–C/N bonds (Pd–C: 1.996 Å) and more labile Fe–N bonds enable rapid transformation (∼1 h), while in the Pt framework, shorter Pt–C/N bonds (Pt–C: 1.990 Å) and a more rigid lattice slow the process (∼24 h). Thermogravimetric analysis indicates subtle differences in network cohesion and bond strength, influencing both thermal and chemical stability.

The irreversible frameworks **2** and **3** were further characterized by IR, TGA, and magnetic measurements. ATR-IR spectra confirm the disappearance of pyrazine vibrations and the appearance of NH_3_ bands (Figure S13). TGA shows initial weight losses of ∼10% for **2** and ∼8% for **3** below 100 °C, consistent with two lattice water molecules. Both frameworks exhibit lower thermal stability than the parent materials, decomposing at ∼180 °C (**2**) and ∼160 °C (**3**). Magnetic susceptibility measurements reveal χ_M_ *T* values of ∼0.4 cm^3^ K mol^–1^ at 300 K, indicating that nearly all Fe­(II) centers adopt low-spin states and remain diamagnetic up to 400 K. Collectively, these results establish that NH_3_-substituted frameworks **2** and **3** retain crystallinity while displaying structural, optical, and electronic properties distinct from the parent **1-Pd** and **1-Pt** frameworks. Table S4 summarizes the reversible and irreversible NH_3_-induced transformations of the Pd and Pt frameworks, correlating guest interaction modes with color changes, kinetics, and spin-crossover behavior.

### Host–Guest Interactions

To probe the specificity of the ammonia-induced transformation, the reactivity of both **1-Pd** and **1-Pt** was investigated against other polar analytes, including water, methanol, and pyridine. The results reveal that the frameworks are not inert but instead exhibit a rich, guest-dependent host–guest chemistry. Short or prolonged exposures of **1-Pd** to water, methanol, and pyridine vapors lead to the formation of the corresponding clathrate compounds: **1-Pd@H**_**2**_ **O**, **1-Pd@CH**_**3**_ **OH**, and **1-Pd@py**, which have been fully characterized as shown in the experimental section and Supporting Information (Figure S16). The analogues **1-Pt@H**_**2**_ **O**, **1-Pt@CH**_**3**_ **OH**, and **1-Pt@py** had previously been characterized,^19,23^ but we reproduced their synthesis following the same experimental conditions we employed in the palladium analogues. Complete desorption of the guest molecules and the concomitant reversible transformation to **1-Pd** and **1-Pt** occur when the clathrates are heated to 160 °C for 24 h.

Powder X-ray diffraction patterns after prolonged exposure to these analytes show that the frameworks are structurally dynamic (Figure 4). Since the crystal structures of **1-Pd** and **1-Pt** are very similar, their PXRD do not differ so much. For the smaller guest molecules, water and alcohols, the pattern is slightly modified above 15° compared with **1-Pd** and **1-Pt.** In contrast, for **1-Pd@py** and **1-Pt@py**, new bands appear in the low-angle region, while above 12° the pattern is very similar across all clathrates.

**Figure 4:**
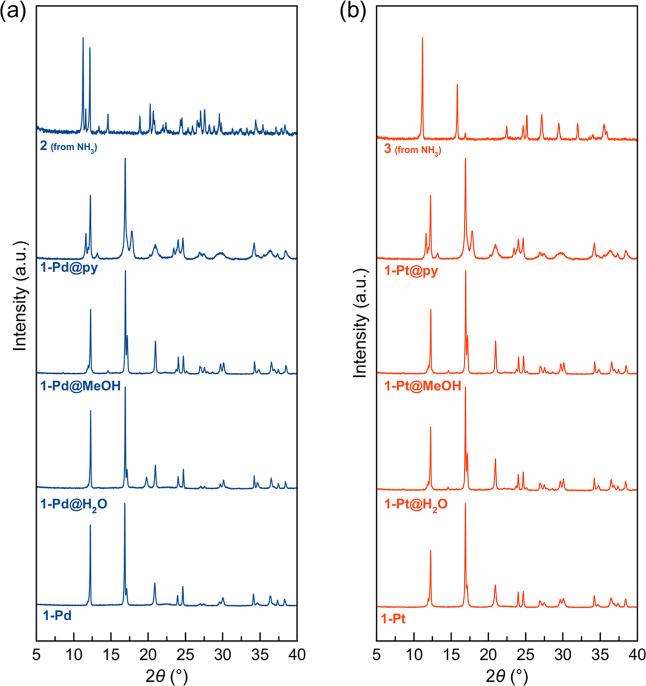
PXRD patterns demonstrating the unique chemical selectivity of (a) the **1-Pd** framework and (b) the **1-Pt** framework. Exposure to H_2_O, MeOH, and pyridine results in the formation of new, distinct clathrate phases. In contrast, only ammonia induces a complete and irreversible structural transformation to the new crystalline phase **2** (Pd) or **3** (Pt).

These PXRD pattern coincidences indicate subtle structural modifications upon guest inclusion, while maintaining the host structures in **1-Pd** and **1-Pt**. This behavior is characteristic of reversible physical guest inclusion. The FT-IR spectra corroborate these findings, showing the presence of guest molecules within the pores while the pyrazine vibrational modes remain intact (Figure S15).

Apparently, only NH_3_ displaces the coordinated pyrazine molecules of the frameworks, hence, transforming the clathrates **1-Pd@NH**_**3**_ and **1-Pt@NH**_**3**_ into the new chemical compounds **2** and **3**. Indeed, the PXRD patterns of **2** and **3** are singular, and compatible with two-dimensional structures made up of [Fe-NC-Pd­(Pt)]_∞_ two-dimensional planes where the Fe­(II) coordination sphere is completed by the ammonia molecules occupying the axial positions as observed in Hofmann type clathrates {[M­(NH_3_)_2_Ni­(CN)_4_]@G}.^27,28^

In this regard, the chemical selectivity of the frameworks **1-Pd** and **1-Pt** for ammonia fixation is evidenced. This comparative study allows for a clear distinction between the two types of selectivity. The framework exhibits physical selectivity, forming different clathrate structures depending on the guest. More importantly, they exhibit chemical selectivity, in which only ammonia exhibits the unique reactivity to induce a permanent, destructive, and reconstructive transformation of the framework via ligand displacement. While the frameworks retain long-range crystallinity, the transformation is not single-crystal-to-single-crystal (SCSC).^29–32^ The porous structure of the Hofmann-type frameworks allows gaseous NH_3_ to access the Fe­(II) coordination sites, where its strong Lewis basicity competes effectively with pyrazine, promoting ligand substitution. This process partially disrupts the single-crystal quality, preventing single-crystal X-ray analysis of the products. Consequently, the proposed structural models for **2** and **3** are based on combined PXRD, IR, TGA, magnetic, and elemental analyses and should be regarded as experimentally supported representations rather than definitive crystallographic solutions. These observations highlight that ammonia induces a selective, crystallinity-retaining transformation through ligand substitution, without implying a true SCSC mechanism.

### Magnetic Properties and Magneto-Structural Correlations

To elucidate the physical consequences of the guest-dependent interactions and irreversible chemical transformations, the magnetic properties of all parent, clathrate, and transformed phases were investigated (Figure 5).

**Figure 5:**
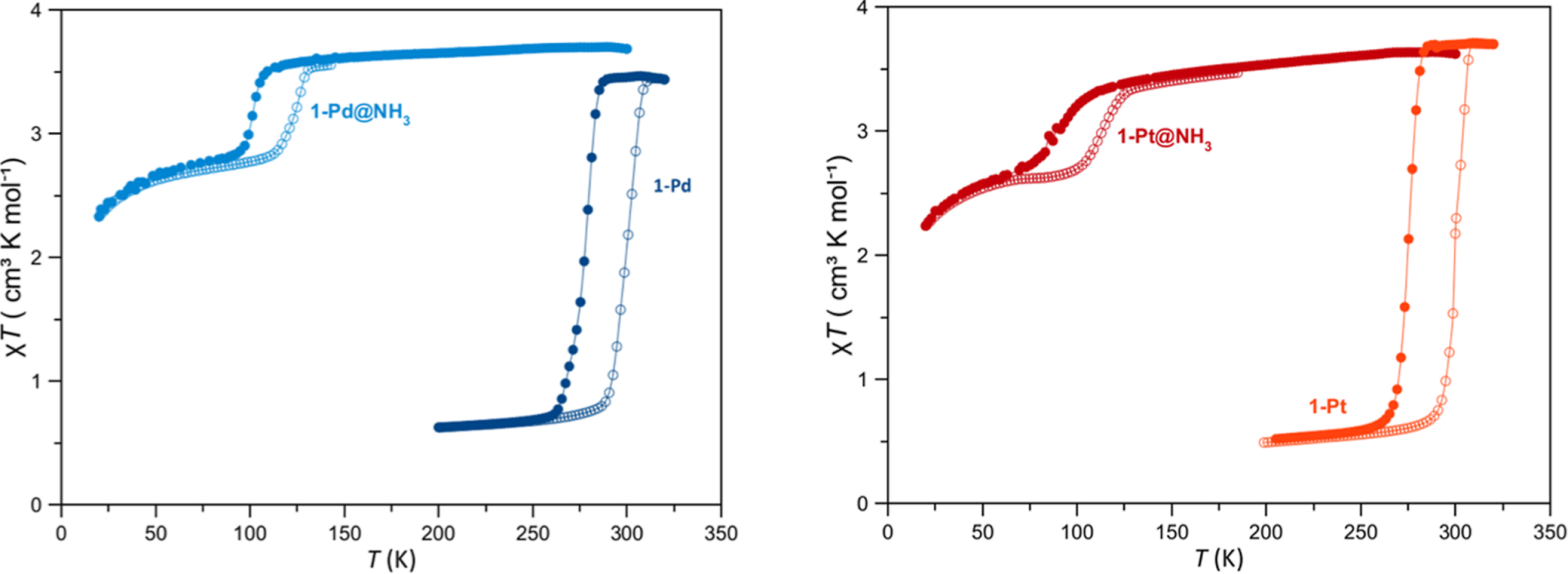
Temperature dependence of the χ_M_ *T* (where χ_M_ stands for the molar magnetic susceptibility and *T* the temperature) measured at 2 K/min under a 10 kOe field for pristine **1-Pd** and **1-Pt** and the ammonia clathrates, **1-Pd@NH**_**3**_ and **1-Pt@NH**_**3**_. Filled and open symbols represent cooling and warming modes, respectively.

The magnetic properties of the frameworks **1-Pd** and **1-Pt** are dramatically altered by the presence of ammonia in the porous structure. As shown in Figure 5, SQUID magnetometry measurements reveal that guest-free frameworks **1-Pd** and **1-Pt** undergo an abrupt, complete, and reversible spin transition from the HS to the LS state upon cooling. The room-temperature χ_M_ *T* value of ∼3.3 cm^3^ K mol^–1^, where χ_M_ stands for the molar magnetic susceptibility and *T* the temperature, is typical for HS Fe­(II). In comparison, the value drops to near zero at 260 K, confirming a complete transition to the diamagnetic LS state for both compounds. The transition exhibits a distinct thermal hysteresis of 21 K­(**1-Pd**) and 25 K (**1-Pt**), with critical temperatures of [**1**-**Pd**: *T*_c_↓ = 277 K and *T*_c_↑ = 298 K; **1-Pt**: *T*_c_↓ = 275 K and *T*_c_↑ = 300 K].^19^

Upon inclusion of ammonia inside the porous structure, the magnetic behavior of **1-Pd**@NH_3_ is profoundly different in comparison to **1-Pd**. The spin transition is shifted by more than 150 K to cryogenic temperatures, with *T*_c_↓ ≈ 105 K and *T*_c_↑ ≈ 128 K, accompanied by a similar thermal hysteresis width of 23 K. The χ_M_ *T* value at 100 K remains high at approximately 2.3 cm^3^ K mol^–1^, which denotes a residual HS fraction of about 50%. At around 100 K, the spin transition becomes kinetically blocked, locking about 50% of the Fe­(II) ions in the HS state. The further decrease in magnetic susceptibility below 20 K can be attributed to the zero-field splitting of the Fe­(II) ions remaining in the HS state. **1-Pt@NH**_**3**_ undergoes an incomplete spin conversion on lowering temperature, with *T*_c_↓ ≈ 105 K and *T*_c_↑ ≈ 128 K as the characteristic temperatures. Contrarily, compounds **2** and **3** adopt the LS configuration in the 400–10 K temperature interval (Figure S12).

The displacement of the spin transition temperature of the Fe­(II) ions down to 100 K in **1-Pd@NH**_**3**_ and **1-Pt@NH**_**3**_ is the result of interactions between the framework and the guest molecules, a phenomenon that has yet to be well documented for **1-Pt**@guest.^19–24^ It has been demonstrated that proximate guest molecules strongly perturb the crystal field strength felt by the Fe­(II) centers. Depending on the guest size and its interaction via hydrogen bonding or π–π interactions with the framework, both stabilization of the HS (*T*_c_ displaced down in temperature) or LS states (*T*_c_ displaced up in temperature), or even inhibition of the spin transition, has been reported. Like water, alcohols, or benzene, the ammonia molecules constrained in the lattice favor locking the Fe­(II) centers in the HS state. Switching to the LS state implies a shortening of the Fe–N bond distances and hence a diminution of the pore size and void space. Guests like benzene, pyridine, and even large amounts of water per iron­(II) ion (up to 5 molecules) inhibit the contraction of the framework to the LS state. This provides a clear microscopic rationale for macroscopic magnetic observation. Here it has been evidenced that the framework **1-Pd** exhibits a very similar host–guest chemistry than **1-Pt** does. However, slight differences appear in regard with the critical temperatures of the spin transition among the palladium and platinum clathrates, which are connected both to the covalency of the Fe-NC bond and the chemical pressure imposed by the frameworks themselves.

## Conclusion

The Hofmann-type heterometallic frameworks **1-Pd** and **1-Pt** display a pronounced dual-mode response to ammonia, combining reversible guest inclusion with irreversible ligand substitution. Short NH_3_ exposure converts the orange frameworks into pale-yellow clathrate phases (**1-Pd@NH**_**3**_ and **1-Pt@NH**_**3**_), preserving the parent structure, while spin-crossover behavior is strongly modulated, with transition temperatures shifted by up to 150 K and partial stabilization of the high-spin state. In contrast, prolonged exposure induces irreversible, crystallinity-retaining transformations in which Fe­(II)-bound pyrazine ligands are replaced by NH_3_, forming red, diamagnetic phases [Fe­(NH_3_)_2_M­(CN)_4_]·2H_2_O (M = Pd (**2**), Pt (**3**)). This ligand substitution proceeds through cleavage of Fe–N­(pyrazine) bonds rather than metal–cyanide bonds, highlighting the bond-specific nature of framework chemical lability.

The transformation kinetics are strongly metal-dependent: the Pd framework reacts within ∼1 h, facilitated by slightly longer Pd–C/N bonds (Pd–C: 1.996 Å) and more labile Fe–N bonds, whereas the Pt analogue converts over ∼24 h, consistent with slightly shorter Pt–C/N bonds (Pt–C: 1.990 Å)^19^ and a more rigid lattice. These differences underscore that framework rigidity and metal–ligand covalency, rather than thermal stability alone, govern ligand substitution rates—the lower thermal decomposition temperature of **1-Pt** compared to **1-Pd** reflects different lattice factors influencing thermal versus chemical reactivity.

Comparative studies with other polar guests reveal both physical and chemical selectivity: only ammonia induces permanent ligand substitution, while water, alcohols, and pyridine produce exclusively reversible clathrate formation. Overall, these results establish clear correlations between guest Lewis basicity, framework dynamics, and spin-crossover behavior, demonstrating how dual-mode host–guest interactions can be harnessed to precisely tune structural, electronic, and magnetic properties in heterometallic coordination frameworks.

## Experimental Section

### Materials

Ammonium iron­(II) sulfate hexahydrate (Mohr’s salt, (NH_4_)_2_Fe­(SO_4_)_2_·6H_2_O, 99%), pyrazine (pz, 99%), potassium tetracyanopalladate­(II) (K_2_[Pd­(CN)_4_], 99.9%), potassium tetracyanoplatinate­(II) hydrate (K_2_[Pt­(CN)_4_]·*x*H_2_O, 99.9%), and ammonium hydroxide (NH_3_, 28% aq.) were purchased from commercial sources and used as received. All solvents were of analytical grade; Milli-Q water and methanol (MeOH, ≥99.8%) were used. **Caution!** Cyanometallate salts are toxic and should be handled with appropriate personal protective equipment.

### Synthesis of [Fe­(pz)­Pd­(CN)_4_]·2H_2_O (1-Pd@H_2_O)

#### Polycrystalline Powder

Under an argon atmosphere, solid pyrazine (102 mg, 1.27 mmol) was added to a stirred solution of (NH_4_)_2_Fe­(SO_4_)_2_·6H_2_O (500 mg, 1.27 mmol) in 50 mL of degassed Milli-Q water. After 5 min, a solution of K_2_[Pd­(CN)_4_] (368 mg, 1.27 mmol) in 50 mL of degassed water was added dropwise (ca. 1 drop per 6 s). The resulting orange-yellow precipitate was stirred for an additional 30 min, filtered, washed with Milli-Q water and methanol, and dried in a desiccator. Yield: 78%. Anal. Calcd for C_8_H_8_FeN_6_O_2_Pd: C, 25.12; H, 2.11; N, 21.97. Found: C, 25.36; H, 2.15; N, 22.27. FT-IR (ATR, cm^–1^): 2170vs [ν­(C≡N)], 1157m, 1125m, 1083m, 1050s, 1027m [ν­(pz)], 803m, 416s. *Single Crystals.* Orange, needle-like single crystals were grown over 3 weeks by slow diffusion of equimolar (0.064 mmol) aqueous solutions of (NH_4_)_2_Fe­(SO_4_)_2_·6H_2_O, pyrazine, and K_2_[Pd­(CN)_4_] in a triple-neck tube.

### Synthesis of [Fe­(pz)­Pt­(CN)_4_]·2H_2_O (**1-Pt@H**_**2**_ **O**)

Polycrystalline powder was synthesized under argon following a modified literature procedure.^18^ An aqueous solution (50 mL) of (NH_4_)_2_Fe­(SO_4_)_2_·6H_2_O (500 mg, 1.27 mmol) and pyrazine (102 mg, 1.27 mmol) was added dropwise to a stirred aqueous solution (50 mL) of K_2_[Pt­(CN)_4_]·*x*H_2_O (479 mg, 1.27 mmol). The orange precipitate was filtered, washed with water and methanol, and air-dried. Anal. Calcd for C_8_H_8_FeN_6_O_2_Pt: C, 20.40; H, 1.71; N, 17.84. Found: C, [20.20%]; H, [1.80%]; N, [18.04%]. FT-IR (ATR, cm^–1^): 2170s [ν­(C≡N)], 1156m, 1131m, 1083m, 1052s, 1025w [ν­(pz)], 802s, 462vs yield: 78%.

### Preparation of Guest-Free Frameworks **1-Pd** and **1-Pt**

The as-synthesized hydrated framework **1-Pd@H**_**2**_ **O** was dehydrated by heating at 160 °C for 24 h to yield the anhydrous framework **1-Pd**. The same procedure was applied to obtain **1-Pt** from **1-Pt@H**_**2**_ **O**.

### Guest Sorption Studies

All sorption experiments were performed using the anhydrous, guest-free frameworks **1-Pd** and **1-Pt**. Samples (ca. 50 mg) were placed in open glass vials inside a 250 mL sealed desiccator containing 20 mL of the volatile liquid (NH_3_(aq, 28%), H_2_O, MeOH, or pyridine). For time-resolved kinetic studies with ammonia, samples of **1-Pd** and **1-Pt** were removed at intervals (1, 3, 10, 30 min, 24 h) for analysis. Reversible clathrates (**1-Pd@NH**_**3**_, **1-Pt@NH**_**3**_) were prepared by 1 min exposure to NH_3_ vapor, followed by 48 h of airing to remove physiosorbed molecules. Other clathrates (**M@H**_**2**_ **O**, **M@MeOH**, **M@py**) were prepared by 48 h exposure. The irreversibly transformed products **2** (Pd) and **3** (Pt) were obtained after >24 h exposure to NH_3_ vapor. Anal. Calcd for: **1-Pd@NH**_**3**_: [Fe­(pz)­Pd­(CN)_4_]·2NH_3_: C_8_H_10_FeN_8_Pd: C, 25.25; H, 2.65; N, 29.45. Found: C, [25.20%]; H, [2.60%]; N, [29.04%]. **1-Pt@NH**_**3**_: [Fe­(pz)­Pt­(CN)_4_]·2NH_3_: C_8_H_10_FeN_8_Pt: C, 20.48; H, 2.15; N, 23.88. Found: C, [20.20%]; H, [2.05%]; N, [23.55%]. **2**: {[Fe­(NH_3_)_2_Pd­(CN)_4_]·2H_2_O}, C_4_H_10_FeN_6_O_2_Pd: C, 14.28; H, 3.00; N, 24.98. Found: C, [14.13%]; H, [3.02%]; N, [24.96%]. **3**: {[Fe­(NH_3_)_2_Pt­(CN)_4_]·2H_2_O}, C_4_H_10_FeN_6_O_2_Pt: C, 11.30; H, 2.37; N, 19.77. Found: C, [11.00%]; H, [2.35%]; N, [19.31%].

### Physical Characterization

Elemental analyses (C, H, N) were performed by the “Servei Central de Suport a la Investigació Experimental” of the Universitat de València. FT-IR spectra were recorded on a Bruker ALPHA II spectrometer (ATR) with 4 cm^–1^ resolution. PXRD patterns were collected at room temperature on a D8 Avance A25 Bruker diffractometer (Cu Kα radiation, λ = 1.54056 Å). TGA was performed on a Mettler Toledo TGA/STDA 851 thermobalance under N_2_ (25–600 °C) at 10 °C/min. DC magnetic susceptibility data were collected on powdered samples (ca. 10–20 mg in a gelatin capsule) using a Quantum Design SQUID magnetometer under a 10.0 kOe field. Diamagnetic corrections were applied for the sample holder and estimated from Pascal’s constants.

### Single-Crystal X-Ray Diffraction (SC-XRD)

X-ray diffraction data for a single crystal of **1** were collected at 294 and 230 K on a [Insert Diffractometer Model, e.g., Agilent SuperNova] diffractometer (Mo Kα radiation, λ = 0.71073 Å). Data were processed using [Insert Software, e.g., CrysAlisPro]. The structure was solved by intrinsic phasing (SHELXT) and refined by full-matrix least-squares on F^2^ (SHELXL). All non-hydrogen atoms were refined anisotropically; H atoms were placed in calculated positions and refined using a riding model. Crystallographic data are summarized in Table S1. Attempts to characterize the guest-loaded phases (**1-Pd@NH**_**3**_, **1-Pt@NH**_**3**_) and transformed products (**2**, **3**) by SC-XRD were unsuccessful, as exposure of single crystals of **1-Pd** and **1-Pt** to ammonia vapor resulted in a loss of diffraction quality. Crystallographic data for the structures of **1-Pd** at 294 and 230 K have been deposited with the Cambridge Crystallographic Data Centre (CCDC) under deposition numbers CCDC [2505843] and CCDC [2505842], respectively. These data can be obtained free of charge via www.ccdc.cam.ac.uk/data_request/cif.

## Supplementary Material



## Notes

Experimental procedures; spectroscopic characterization (FT-IR and TGA); multitemperature single-crystal X-ray diffraction data for 1-Pd (high-spin and low-spin states) and 1-Pd@NH_3_, 2, and 3; powder X-ray diffraction patterns; magnetic measurements (χ_M_T vs T); optical micrographs; host–guest selectivity studies. Crystallographic information files for all single-crystal structures (PDF)
