# Supplementary material for: “Ammonia Induced Framework Transformations and Spin-Crossover in Fe(II) Hofmann MOFs”
Source: Inorg Chem. 2026 Apr 1;65(14):8052–60. doi: 10.1021/acs.inorgchem.6c00626 (PMC13080993; doi:10.1021/acs.inorgchem.6c00626)
Supplement: Supplementary file 1 [file ic6c00626_si_001.pdf]

Supporting Information

"Ammonia Induced Framework Transformations and Spin-Crossover in Fe(II) Hofmann MOFs"

Mario Pacheco,[a,b] Annena Jesuman,[a] Higinio Maqueda-Márquez,[a] Javier González-Platas,[c] Ana Belén Gaspar [a*]

ana.b.gaspar@uv.es

[a] Institut de Ciència Molecular (ICMol)-Departament de Química Inorgànica, Universitat de València, C/ Catedrático José Beltrán 2, 46980 Paterna, Spain.

[b] Departamento de Física-Instituto Universitario de Estudios Avanzados en Física Atómica, Molecular y Fotónica (IUDEA). MALTA Consolider Team. Universidad de La Laguna, Avda. Astrofísico Fco. Sánchez s/n, La Laguna, Tenerife, E-38204, Spain.

[c] Facultad de Química, Universidad de la República, Av. Gral. Flores 2124, 11800 Montevideo, Uruguay.

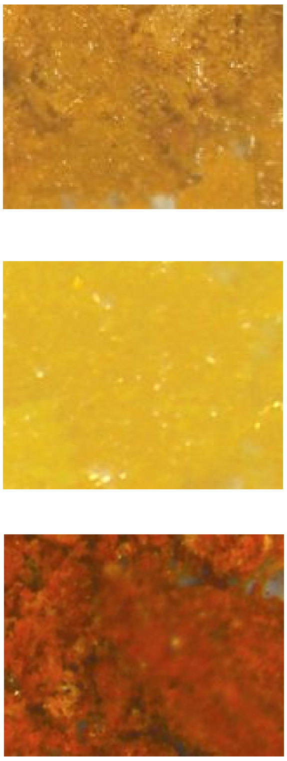

Figure S1. Color of compounds **1-Pd** [**1-Pt**] (top), **1-Pd**@NH_3 [**1-Pt**@NH_3] (middle), **2** and **3** (bottom).

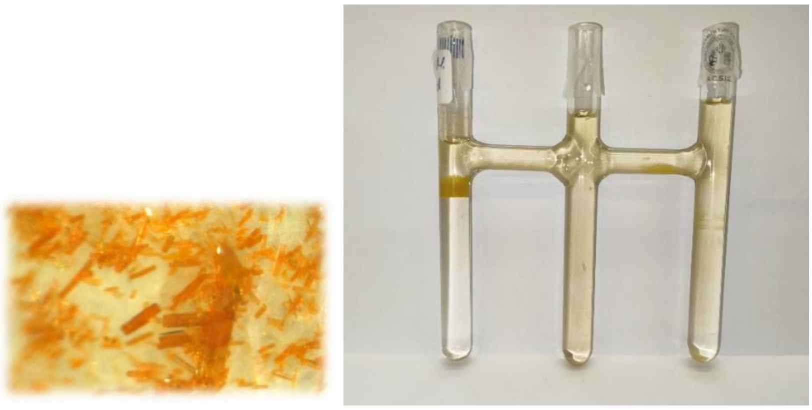

Figure S2. (Left) Optical micrograph of as-synthesized single crystals of $[Fe(pz)Pd(CN)_4]$ (**1-Pd**), showing their orange color and needle-like morphology. (Right) Photograph of the triple-neck tube used for the slow diffusion synthesis.

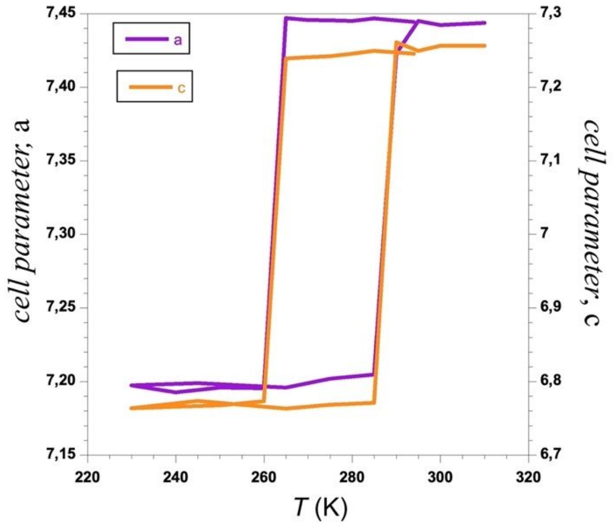

Figure S3. Thermal evolution of the unit cell parameters *a*-*c* (Å) in the cooling and warming modes derived from multitemperature single-crystal X-ray determination in **1-Pd**.

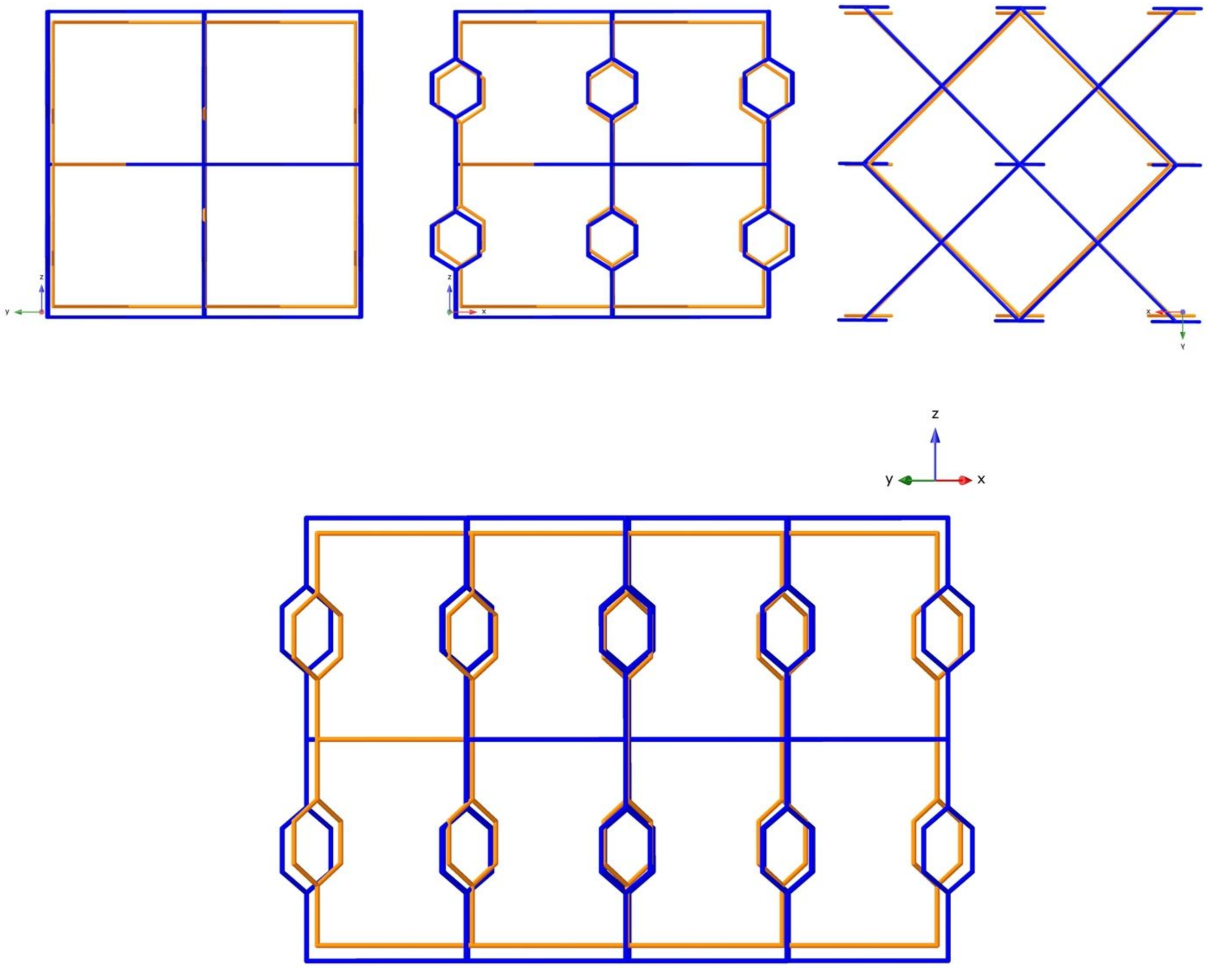

Figure S4. Illustration of the reversible framework contraction/expansion in [Fe(pz)Pd(CN)$_4$] (**1-Pd**) upon spin state transition (HS state, blue) and (LS state, orange).

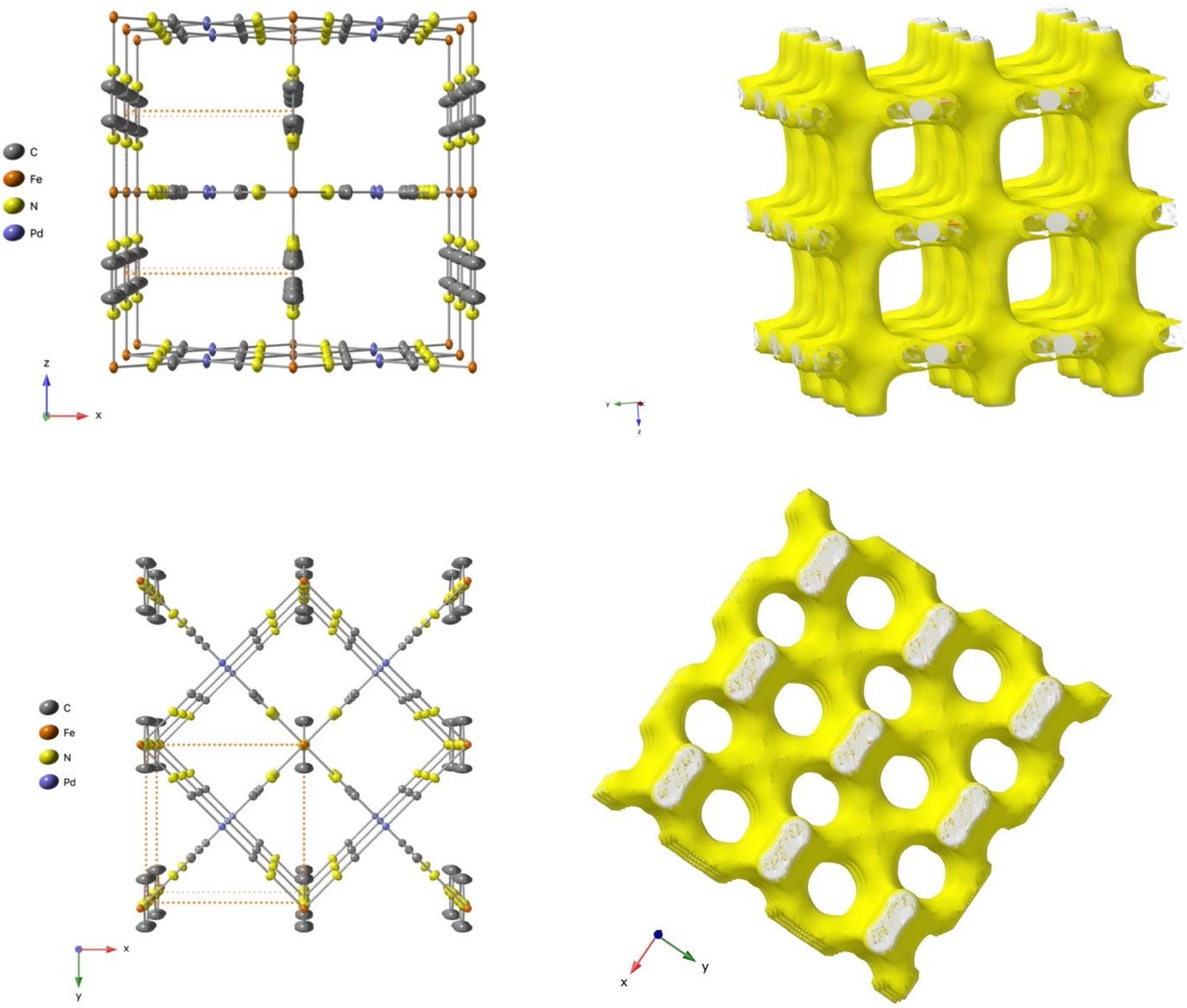

Figure S5. Left: Illustrations of the porous crystalline structure of [Fe(pz)Pd(CN)$_4$] (**1-Pd**) at 294 K (HS state) in the z and y directions. Thermal ellipsoids are shown at the 50% probability level. Right: Pictures emphasizing the void space, cavities, in the structure.

Porosity data	1-Pd (HS, state)	1-Pd (LS, state)
Volume	403.28 Å³	350.36 Å³
Filled space	12.25 Å³ (3.04%) per unit cell	5.30 Å³ (1.51%) per unit cell
Void space	391.03 Å³ (96.96%) per unit cell	345.06 Å³ (98.49%) per unit cell

Table S1. Crystallographic data and structure refinement details for **1-Pd·2H$_2$O** at 294 K (HS) and 230 K (LS).

**Parameter**	**1-Pd·2H$_2$O (HS, High Spin state)**	**1-Pd·2H$_2$O (LS, Low Spin state)**
Chemical formula	$C_8H_8FeN_6O_2Pd$	$C_8H_8FeN_6O_2Pd$
Formula weight	382.45	382.45
Temperature	298(2)	**230.0(5)**
Crystal system	Tetragonal	Tetragonal
Space group	*P* 4/*m m m*	*P* 4/*m m m*
Unit cell dimensions		
a (Å) = b (Å)	7.4484(6)	**7.1973(10)**
c (Å)	7.2691(16)	**6.7635(14)**
α (°) = β (°) = γ (°)	90	**90**
Volume (Å^3)	403.28(10)	**350.36(10)**
Z (Formula units/cell)	1	**1**
Density (calculated) (Mg/m^3)	1.426	**1.813**
Absorption coefficient (mm^{-1})	2.000	**2.321**
F(000)	165.5	**185.5**
Radiation (λ, A)	Mo Kα (0.71073)	Mo Kα (0.71073)
Reflections collected / unique	409 / 346	**804/268**
R_{int}	0.0461	**0.0737**
Parameters refined	22	**22**
Goodness-of-fit on F^2	0.9957	**1.001**
Final R indices [I > 2σ(I)]		
R1	0.0380	**0.0486**
wR2	0.1017	**0.0991**
R indices (all data)		
R1	0.0488	**0.0541**
wR2	0.1091	**0.1013**
Largest diff. peak/hole (e/Å^3)	1.05/-0.84	**3.06/-2.15**
CCDC Deposition No.	[2505843]	[2505842]

Table S2. Comparison of selected bond lengths (Å) and angles (°) for **1-Pd·2H$_2$O** at 294 K (HS) and 230 K (LS).

**Parameter**		**294 K (HS)**	**230 K (LS)**	**Δ (LS - HS)**
**Unit cell axis**	**a**	**7.4443**	**7.1973**	**0.2470**
**Unit cell axis**	**b**	**7.4443**	**7.1973**	**0.2470**
**Unit cell axis**	**c**	**7.2455**	**6.7635**	**0.4820**
	**Bond/Angle**	**Fe(II) Coordination Sphere**		
**Axial Bond**	Fe-N(pz)	2.229	1.972	0.2570
**Equatorial Bond**	Fe-N(CN)	2.123	1.953	0.1700
**Axial-Equatorial Angle**	N(pz)-Fe-N(CN)	90.0	90.0	0.00
	**Framework Linkers (Spectators)**			
**Cyanometallate (Pd)**	Pd-C(CN)	1.996	1.953	0.043
**Cyanometallate (CN)**	C-N(CN)	1.144	1.158	-0.014

Table S3. Thermal evolution of the unit cell parameters *a*-*c* (Å) and volume (Å^3) in the cooling and warming modes derived from multitemperature single-crystal X-ray determination in **1-Pd**.

Temperature, K (cooling mode)	a	c	Volume	Temperature, K (warming mode)	a	c	Volume
294	7,4443	7,2455	401,53	245	7,1989	6,7734	351,03
285	7,4468	7,2495	402,02	265	7,1958	6,7629	350,18
280	7,4450	7,2455	401,60	275	7,2018	6,7680	351,03
275	7,4455	7,2420	401,46	285	7,2046	6,7710	351,47
270	7,4457	7,2407	401,41	290	7,4230	7,2610	400,00
265	7,4470	7,2390	401,40	295	7,4450	7,2492	401,81
260	7,1953	6,7730	350,65	300	7,4421	7,2561	401,88
250	7,1959	6,7673	350,41	310	7,4437	7,2565	402,08
240	7,1926	6,7658	350,02				
230	7,1973	6,7635	350,36				

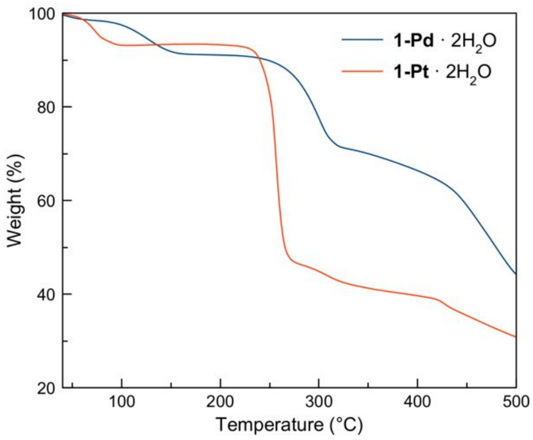

Figure S6. Thermogravimetric analysis (TGA) curves for as-synthesized **1-Pd·2H$_2$O** (blue) and **1-Pt·2H$_2$O** (red) under a N_2 atmosphere. The initial weight loss of ca. 8.9% for **1-Pd·2H$_2$O** (calc. for 2H$_2$O: 9.4%) and ca. 6.8% for **1-Pt·2H$_2$O** (calc. for 2H$_2$O: 7.6%) confirms the presence of two lattice water molecules. The anhydrous frameworks exhibit different thermal stabilities; **1-Pt** begins its primary decomposition at ca. 220 °C, while **1-Pd** is stable up to ca. 260 °C.

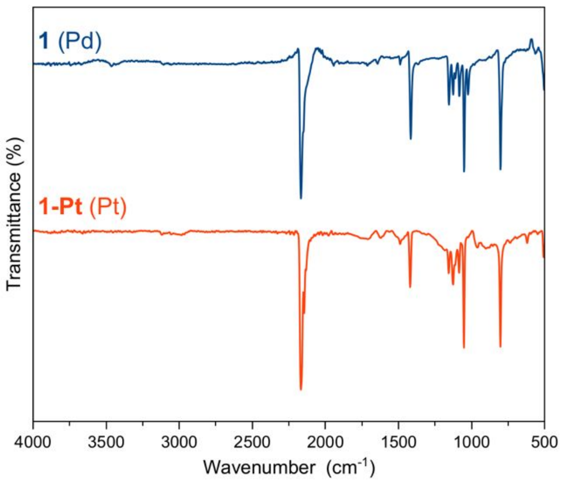

Figure S7. FT-IR (ATR) spectra of the anhydrous frameworks **1-Pd** (Pd, blue) and **1-Pt** (Pt, red). The similarity of the vibrational modes, including the characteristic cyanide stretch (ν(C≡N)) above 2200 cm^{-1} and the pyrazine-related bands, confirms their isostructural nature.

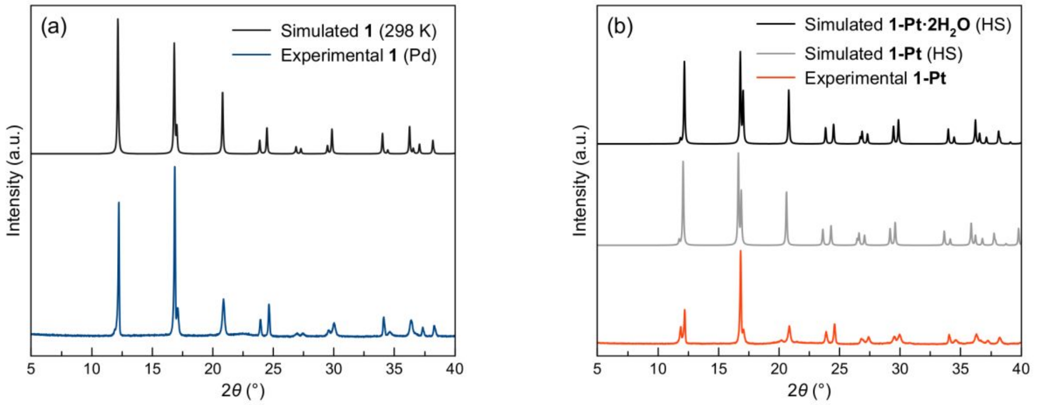

Figure S8. PXRD patterns validating the phase purity, isostructural nature, and rigidity of the pristine frameworks. **(a) Palladium system:** Experimental pattern of bulk anhydrous **1** (blue) shown against the pattern simulated from the single-crystal structure of **1** (black). **(b) Platinum system:** Experimental pattern of bulk anhydrous **1-Pt** (red) shown against the simulated patterns for both anhydrous **1-Pt** (gray) and hydrated **1-Pt·2H$_2$O** (black). The excellent agreement in all cases confirms phase purity. The near-identical patterns in (b) also demonstrate the structural rigidity of the **1-Pt** framework, which does not significantly distort upon water inclusion.

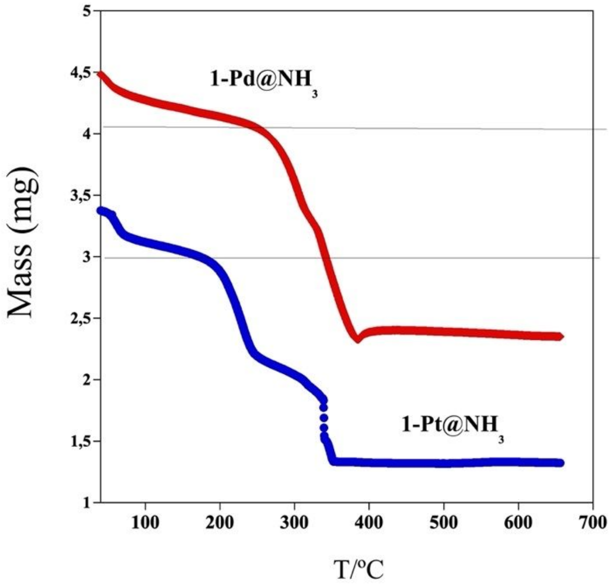

Figure S9. TGA analysis for **1-Pd**@NH_3 and **1-Pt**@NH_3. The weight loss of ca. 10% for **1-Pd@NH_3** (calc. for 2 NH_3: 9.4%) and ca. 8% for **1-Pt**@NH_3 (calc. for 2 NH_3: 7.6%) confirms the presence of two ammonia molecules inside the frameworks. After desorption of guest molecules, the frameworks exhibit different thermal stabilities as observed for pristine frameworks Figure S2; **1-Pt** begins its primary decomposition at ca. 220 °C, while **1-Pd** is stable up to ca. 260 °C.

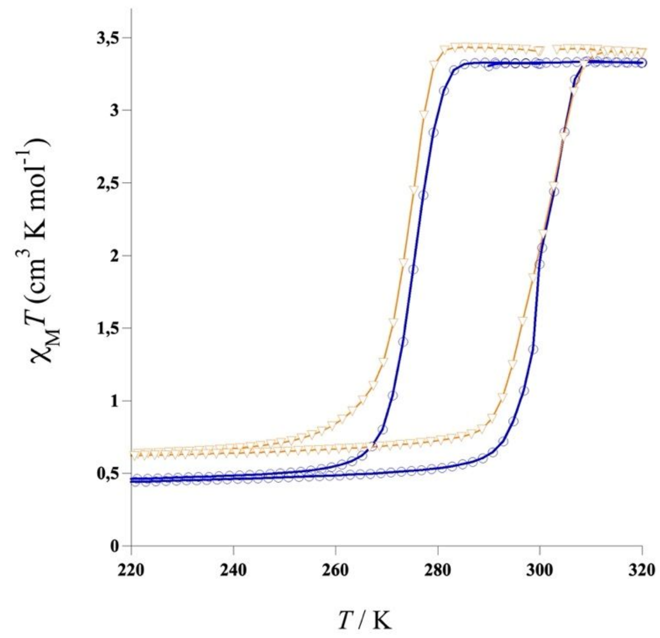

Figure S10. Variable-temperature magnetic susceptibility (χT vs T) of **1-Pd** (orange triangles) and **1-Pt** (blue circles) after NH_3 desorption. The data closely match those of the pristine frameworks, demonstrating that the spin-crossover behavior is fully recovered upon guest removal.

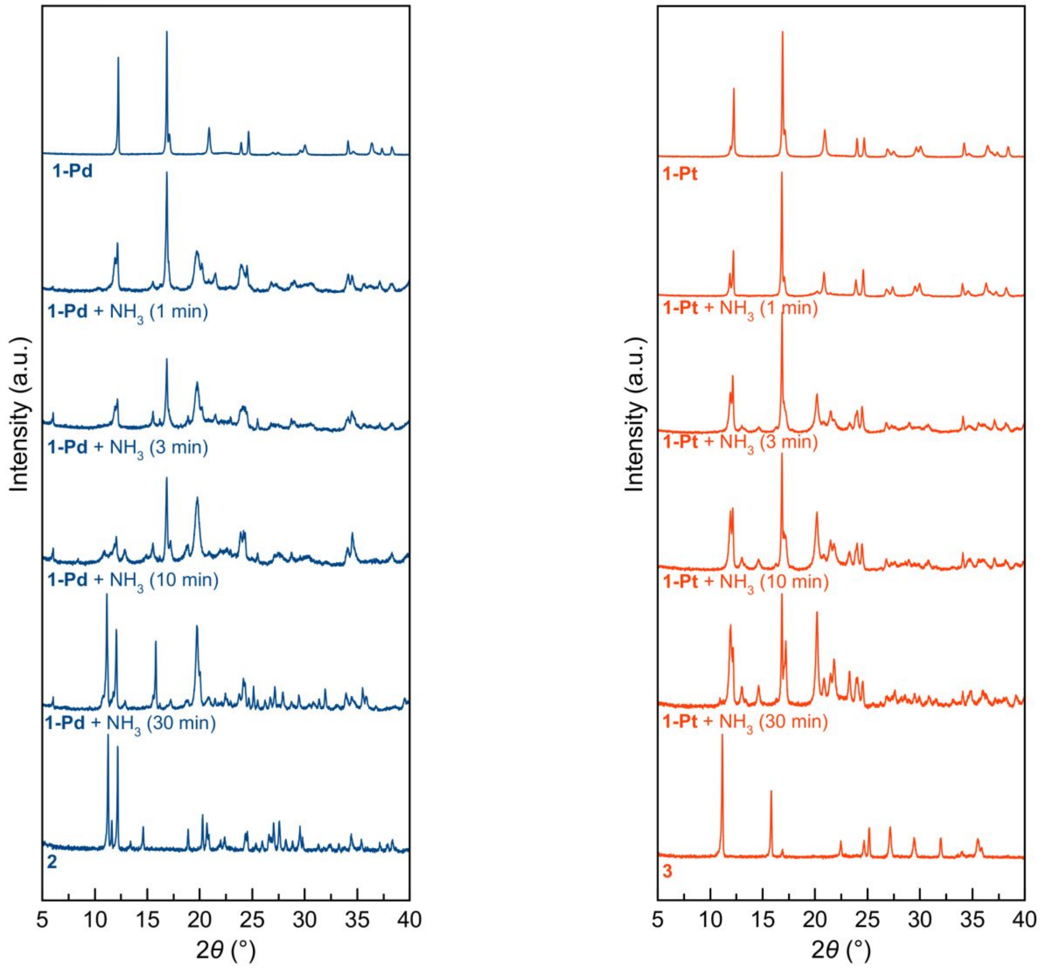

Figure S11. Comparative kinetic study of the irreversible transformation upon exposure to ammonia vapor. Time-resolved PXRD patterns for the **1-Pd** framework (Left) and for the **1-Pt** framework (Right). The palladium system undergoes a rapid conversion to product **2**, which is nearly complete within 30 minutes, evidenced by the fast evolution of new diffraction peaks. In stark contrast, the platinum analogue is kinetically inert on the same timescale, remaining almost unchanged after 30 minutes and only fully converting to product **3** after 24 hours.

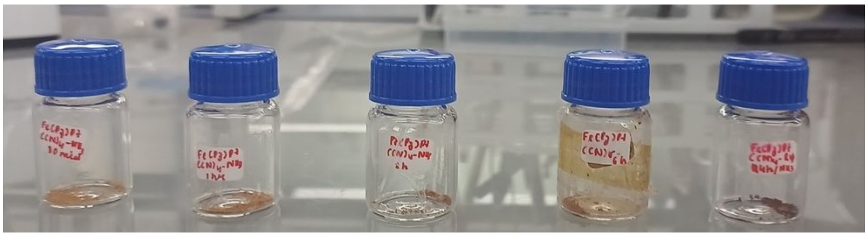

Figure S12. Progressive color change of **1-Pt** upon exposure to NH_3 and transformation into **3**. From left to right: 30 minutes, 1 hour, 2 hours, 6 hours and after 24 hours.

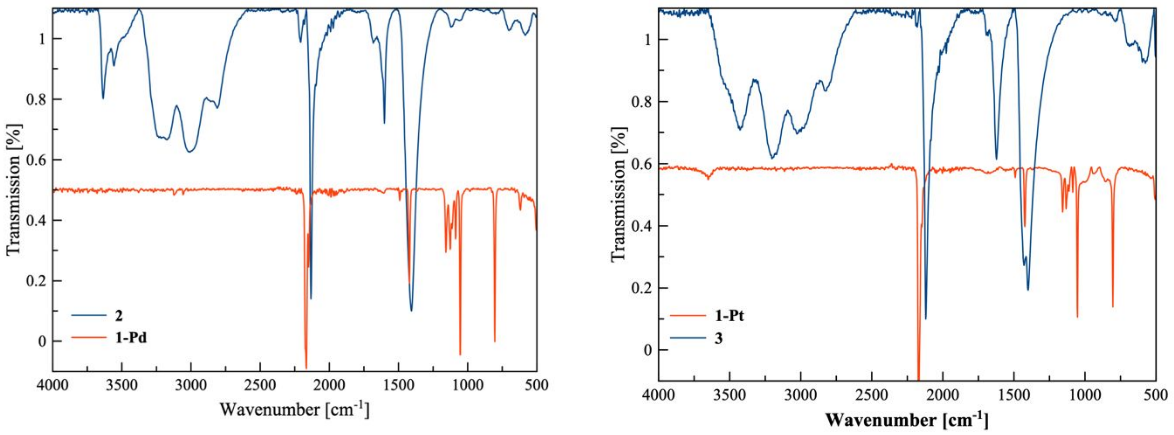

Figure S13. IR spectrum of **2** (Pd) and **3** (Pt) compared to the IR of frameworks **1-Pd** and **1-Pt**.

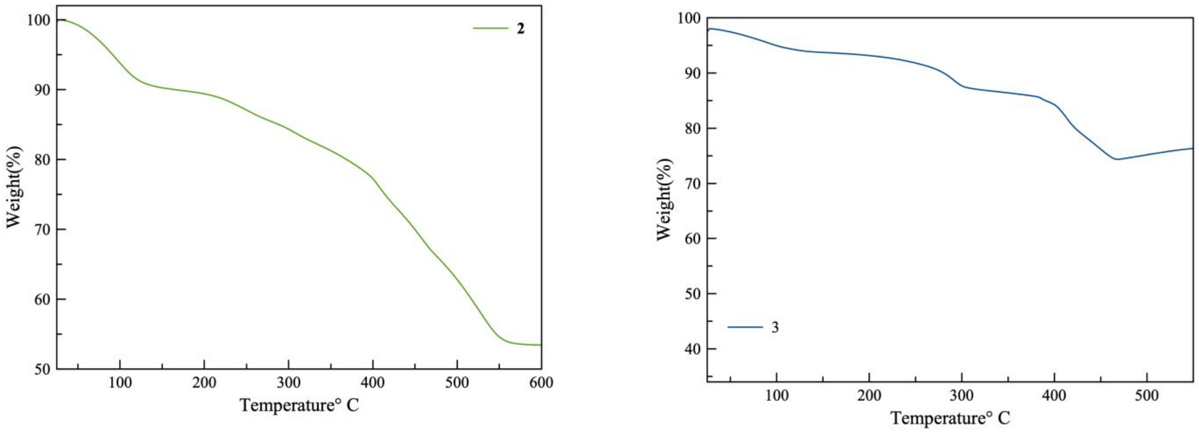

Figure S14. TGA analysis for **2** and **3**. The weight loss of ca. 10% for **2** (calc. for 2 H_2O: 10.7%) and ca. 8% for **3** (calc. 2 H_2O: 8.4%) below 100 ºC confirms the presence of water in the frameworks. After desorption of water molecules, the frameworks exhibit lower thermal stabilities compared to **1-Pd** and **1-Pt**, see for instance Figure S2; **3** begins its primary decomposition at ca. 160 °C, while **2** is stable up to ca. 180 °C. In contrast, **1-Pt** and **1-Pd** decompose at ca. 220 °C and 260 °C, respectively. This is clear evidence that they are different compounds.

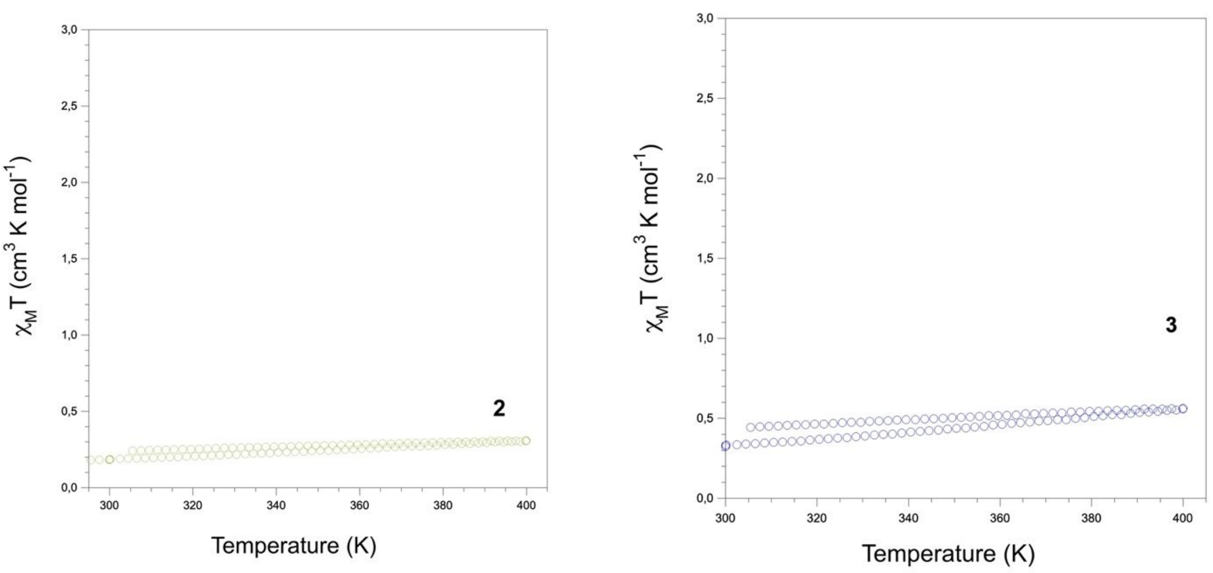

Figure S15. Temperature dependence of the $\chi_M T$ (where χ_M stands for the molar magnetic susceptibility and T the temperature) measured at 2 K/min under a 10 kOe field in the interval of 300 K- 400 K, in the warming and cooling modes, for **2** and **3**. At 300 K, the $\chi_M T$ values of 0.4 cm^3 K mol^{-1} indicates that nearly 100% of the Fe(II) ions are in the LS state. Warming up the samples to 400 K doesn't provoke changes in the magnetic properties.

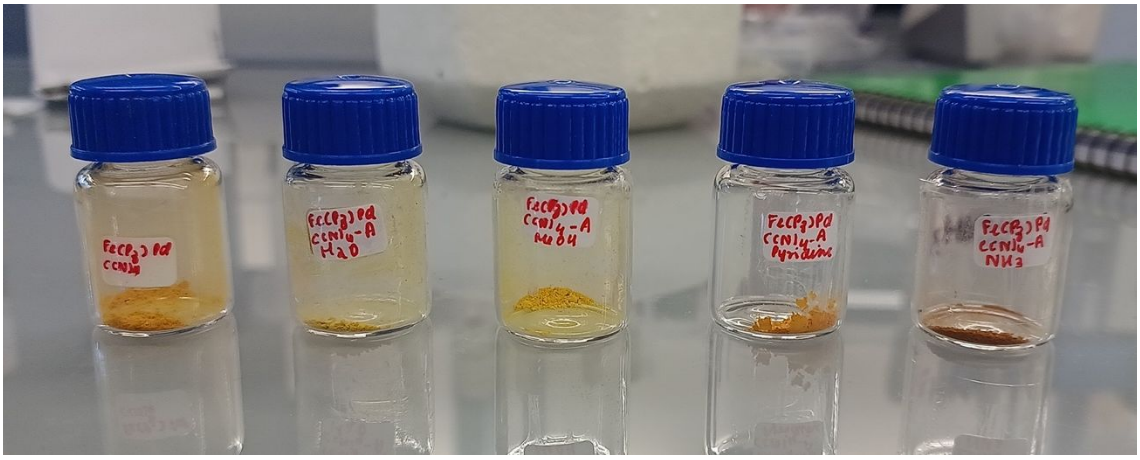

Figure S16. Photographs illustrating the chemical selectivity of the **1-Pd** framework after 48 hours of exposure to water, alcohols, pyridine and ammonia. (From left to right): Pristine anhydrous **1-Pd** (orange), **1-Pd**@H_2O (pale yellow), **1-Pd**@MeOH (orange-yellow), and **1-Pd**@pyridine (orange). While other vapors cause minor, reversible color changes, only ammonia (right) induces a dramatic, irreversible color change to the dark red of the transformed product **2**, demonstrating the unique chemical selectivity for the ligand-displacement reaction.

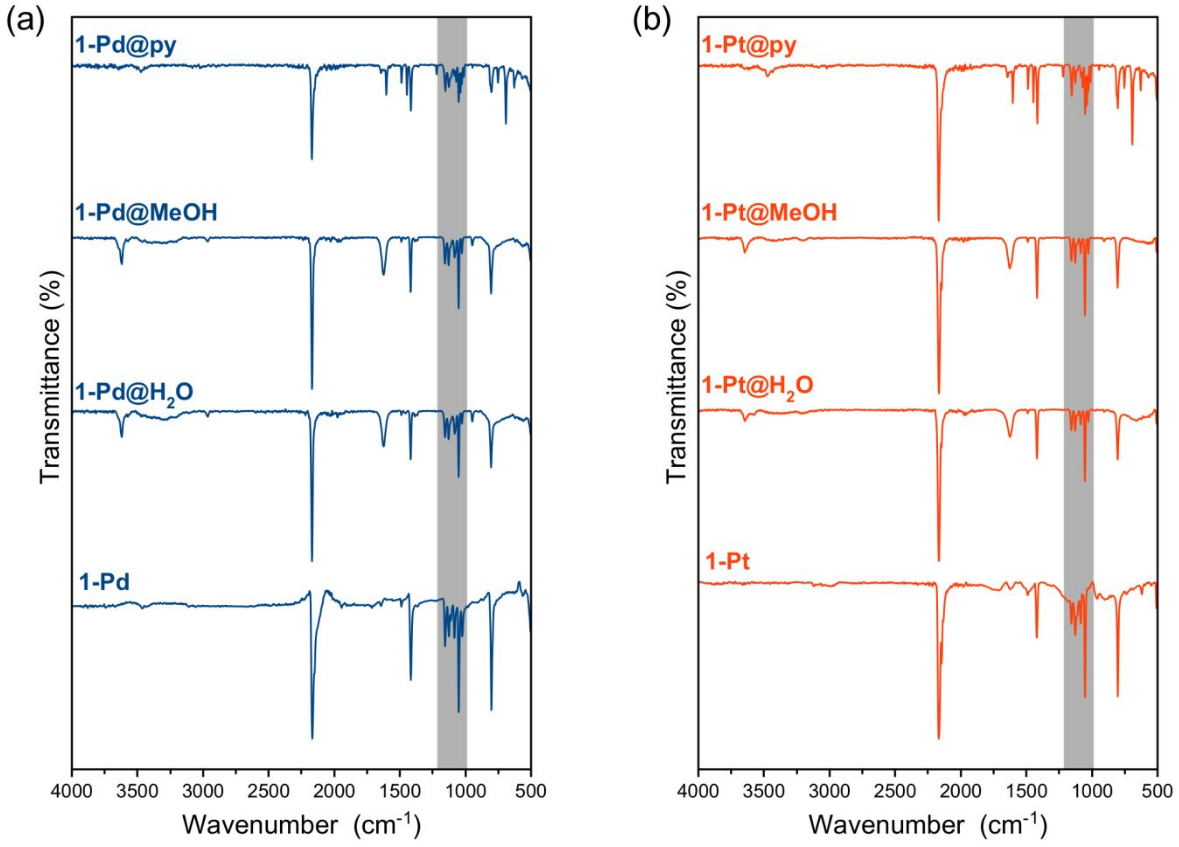

Figure S17. FT-IR (ATR) spectra for the clathrates (a) the 1-Pd framework and (b) the 1-Pt framework. The data confirms that, unlike ammonia, other polar solvents only induce reversible guest inclusion. In all samples exposed to H_2O, MeOH, and Pyridine (py), the characteristic vibrational modes of the pyrazine ligand (highlighted by the gray shaded region, ca. 1000-1150 cm^{-1}) are clearly retained, proving that no irreversible ligand displacement has occurred. This is in direct contrast to the spectra of the ammonia-transformed products (see Figure S13).

Table S4. Summary of optical response, ammonia interaction mode, transformation kinetics, and spin-crossover (SCO) behavior for Pd- and Pt-based Hofmann frameworks. Short NH_3 exposure induces reversible clathrate formation with strong SCO modulation, whereas prolonged exposure leads to irreversible ligand substitution and suppression of spin crossover.

Compound	Guest state	Color	NH_3 interaction mode	Transformation time	SCO behaviour
**1-Pd**	Guest-free	**Orange**	-	-	Abrupt SCO
**1-Pd@NH_3**	Reversible clathrate	**Pale yellow**	Physical inclusion	≤ 1 min (reversible)	SCO strongly modulated
**2**:$[Fe(NH_3)_2Pd(CN)_4]\cdot 2H_2O$	Ligand-substituted	**Red**	Chemical substitution	~1 h (irreversible)	SCO suppressed
**1-Pt**	Guest-free	**Orange**	-	-	Abrupt SCO
**1-Pt@NH_3**	Reversible clathrate	**Pale yellow**	Physical inclusion	≤ 1 min (reversible)	SCO strongly modulated
**3**:$[Fe(NH_3)_2Pt(CN)_4]\cdot 2H_2O$	Ligand-substituted	**Red**	Chemical substitution	~24 h (irreversible)	SCO suppressed
